# Pathogenic fungi-induced susceptibility is mitigated by mutual *Lactobacillus plantarum* in the *Drosophila melanogaster* model

**DOI:** 10.1186/s12866-019-1686-1

**Published:** 2019-12-21

**Authors:** Wanzhen Su, Jialin Liu, Peng Bai, Baocang Ma, Wei Liu

**Affiliations:** 1 Department of Clinical Medical, Shanxi Medical University Fenyang College, Fenyang, 032200 Shanxi China; 2 Department of Basic Medical, Shanxi Medical University Fenyang College, Fenyang, 032200 Shanxi China; 3 Department of Medical Laboratory Science, Shanxi Medical University Fenyang College, Fenyang, 032200 Shanxi China

**Keywords:** *L. plantarum*, Fungal infection, *Drosophila*, Antagonist, Oviposition

## Abstract

**Background:**

Since animals frequently encounter a variety of harmful fungi in nature, their ability to develop sophisticated anti-fungal strategies allows them to flourish across the globe. Extensive studies have highlighted the significant involvement of indigenous microbial communities in human health. However, the daunting diversity of mammalian microbiota and host genetic complexity are major obstacles to our understanding of these intricate links between microbiota components, host immune genotype, and disease phenotype. In this study, we sought to develop a bacterium-fungus-*Drosophila* model to systematically evaluate the anti-fungal effects of commensal bacteria.

**Results:**

We isolated the pathogenic fungal strain, *Diaporthe* FY, which was detrimental to the survival and development of *Drosophila* upon infection. Using *Drosophila* as a model system, *Drosophila*-associated *Lactobacillus plantarum* functioned as a probiotic, and protected the flies from mortality induced by *Diaporthe* FY. Our results show that *L. plantarum* hindered the growth of *Diaporthe* FY in vitro, and decreased the mortality rate of *Diaporthe* FY-infected flies in vivo, consequently mitigating the toxicity of *Diaporthe* FY to the hosts. Additionally, the presence of *L. plantarum* overrode the avoidance of oviposition on *Diaporthe* FY-associated substrates.

**Conclusions:**

*Diaporthe* FY was identified as a potential *Drosophila* pathogen*.* Commensal *L. plantarum* mitigated the susceptibility of *Drosophila* to pathogenic fungi, providing insight into the natural interplay between commensal and pathogenic microbial communities that contribute to animal health and pathogenesis.

## Background

Metazoans harbor a plethora of indigenous microbes (collectively referred to as the microbiota) that routinely influence the physiology and fitness of their host [1, 2], while in turn, the hosts shape the gut microbiota. This forging symbiosis enables the hosts to outcompete a variety of pathogens in the environment. In fact, several host phenotypes are shaped largely by the combination of the genome and microbiome [3, 4]. Consequently, commensals are critically linked to host fitness, including development, fecundity, and lifespan. However, host phenotypes have been traditionally assessed in the context of their microbiota, with little attention devoted to effects of the microbiota on host fitness. In particular, the underlying mechanisms by which the microbiota assist their hosts in combating pathogens remain largely undefined.

In the wild, *Drosophila* mainly feed and breed on rotting fruits that are inhabited by both mutualistic and antagonistic microbes [5, 6]. Due to their saprophagous foraging behavior, *Drosophila* ingest many potentially pathogenic fungi from either food resources or the surrounding environment [7, 8]. Although most microbes are not pathogenic [9], pathogens indeed engender the occurrence and severity of infection in the fly. Antagonistic fungi generate an astonishing variety of secondary metabolites that threaten insect life [10–12]. In addition, plant thorn injury and ectoparasitic mite biting frequently result in cuticle breaches, which aggravates the fungal infection. However, flies fundamentally employ antifungal strategies to flourish in the wild [7]. Although extensive studies have revealed that the microbiota promote the immune response to pathogenic fungi development and restrict pathogen colonization [2, 13, 14], the role of specific bacterial species and/or strains in combating pathogens remain poorly understood. Therefore, there is a need for a model organism to examine the intricate interconnection between hosts, commensals, and pathogens.

*Drosophila* frequently acquires commensals through plant food, and provides amenable insight into the mechanisms by which commensals outcompete pathogens, due to genetic tractability and the ease of generating gnotobiotic animals [15, 16]. The *Lactobacillus* genus is one of most common bacteria present in appreciable numbers in both mammals and *Drosophila* [6]. Moreover, studies have shown that *L. plantarum* fully recapitulates the beneficial effects of the complex microbiota, and influences several aspects of host physiology, including behavior, gut epithelial homeostasis, nutrition, and postembryonic development [13, 17–19]. Moreover, *L. plantarum* is required to protect flies against fly infection induced by food-borne bacteria (e.g., *Pectobacterium carotovorum*) [20]. Current molecular and genomic studies highlight the opportunities and challenges to uncover the interactions of entomopathogenic fungi and fly hosts. However, it remains unknown whether *L. plantarum* can protect hosts from pathogenic fungal infections.

To address these biological questions, we developed a *Drosophila* /bacterium/fungus ecological system that afforded the examination of commensal antagonism against pathogenic fungi. We found that commensal *L. plantarum* mitigated the pathogenic fungi-induced susceptibility of *Drosophila*, providing insight into the ecological significance that commensal bacteria may represent an integral contributor to *Drosophila* fitness upon infection.

## Results

### Isolation and identification *Drosophila*-associated fungi

**Fig. 1:**
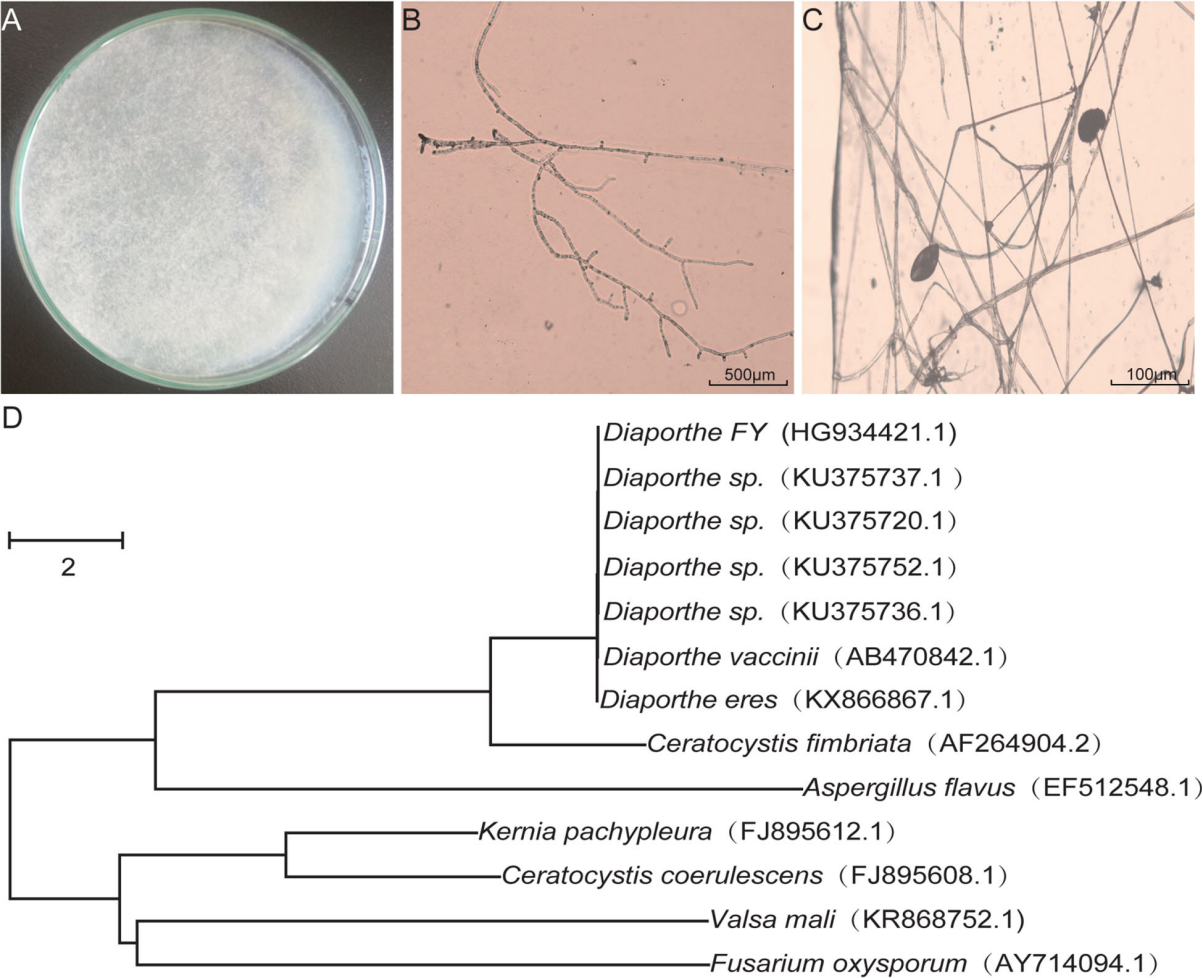
Morphologies and phylogenetics of *Diaporthe* FY (**a**) Colony growth on the yeast-potato medium. Image depicts fungal development after 48 h incubation at 25 °C on nutrient-rich medium. **b** and **c** Mycelia, conidiophores, and conidia of *Diaporthe* FY. **d** The phylogenetic tree of *Diaporthe* FY and its homologs constructed using the neighbor-joining method. Bar: Nucleotide divergence; numbers in the notes present bootstrap percentages; numbers in parentheses are the GenBank accession

A strain of *Drosophila*-associated fungus was isolated from fly food with mold in accordance with standard protocols. This strain was typically a filamentous fungus with white, velvety-like mycelia and dark grey conidial masses (Fig. 1a and b). The conidiophores were bi-verticillate with smooth-walled stipes, bearing short conidial chains (Fig. 1c). It promptly grew at the optimal temperature of 28 °C - 30 °C and ramified the plates (Φ = 90 mm) within 48 h. To confirm the reliability of the morphological identification, the strain was subjected to molecular identification based on an rDNA ITS sequence analysis. Based on the BLASTn search, it displayed > 99% similarity with a published sequence of *Diaporthe sp.* (identities = 555/558) and was relatively close to other *Diaporthe* members. To distinguish our isolate from other strains, it was henceforth termed, *Diaporthe* FY. For taxonomic reconstruction, the other 12 sequences, including out-group species, were retrieved from GenBank to generate a phylogenetic tree (Fig. 1d). *Diaporthe* species are among the most frequent endophytes of a wide-range of plants, including grapevines [21, 22]. Due to the saprophytic foraging behavior, flies might ingest *Diaporthe* from either food or surrounding resources.

### *Diaporthe* FY is a potential pathogen of *D. melanogaster*

**Fig. 2:**
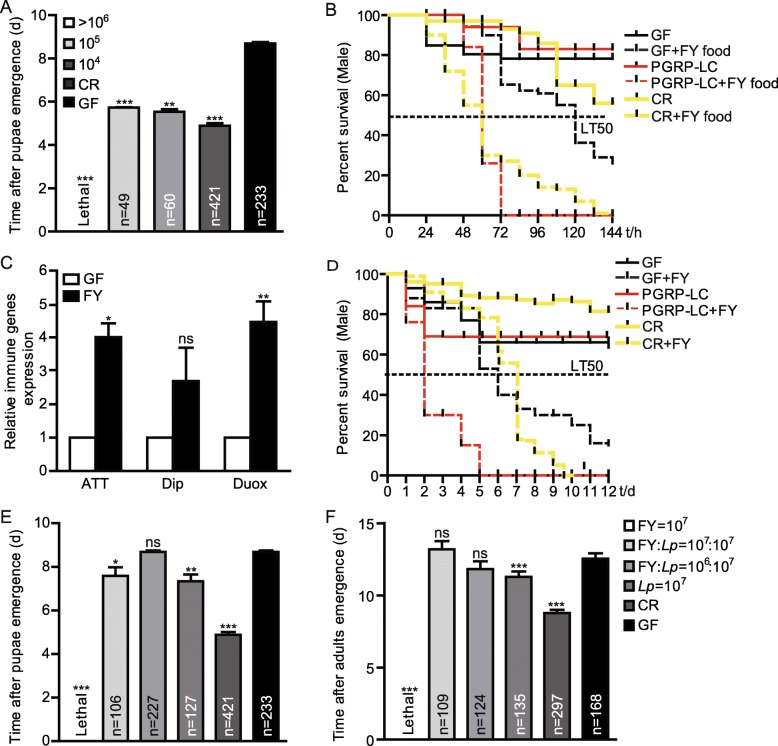
Commensals alleviate the toxicity of *Diaporthe* FY to hosts. **a** The timing of pupa formation of the flies associated with *Diaporthe* FY. GF larva were inoculated with mixed bacteria, sterile PBS, or *Diaporthe* FY to generate CR, GF, and *Diaporthe* FY-associated flies, respectively. Pupae formation was counted daily. **b** The survival of male adults fed *Diaporthe* FY molds in food. Fly food was inoculated with 10^8^ *Diaporthe* FY spores, and incubated at 25 °C for 48 h. Male adults of conventionally reared- and germ-free wild-type and conventionally reared- *PGRP-LC* mutants were orally infected by feeding with *Diaporthe* FY, and the number of dead flies was calculated. **c** *Diaporthe* FY triggered the innate immune response. RT-qPCR analysis of the gut showed that the relative levels of ATT, Dip, and Duox expression were increased upon *Diaporthe* FY infection (*n* = 3). **d** The survival rate of conventionally reared- and germ-free wild-type and *PGRP-LC* mutant male adults infected with *Diaporthe* FY. Male adults were septically infected by punching flies with *Diaporthe* FY spores, and the number of dead flies was calculated. **e** and **f** *L. plantarum* attenuated the toxicity of *Diaporthe* FY to flies. The timing of pupa formation and adult eclosion was assessed in the presence of *Diaporthe* FY, *L. plantarum,* or the mixture of both, respectively. A one-sample *t*-test; * *P* < 0.05; ** *P* < 0.01

*Drosophila* frequently encounter a variety of commensal or pathogenic microbes in the wild. We first asked whether *Diaporthe* FY was beneficial or detrimental to *Drosophila*. To address this question, we examined the developmental timing and survival rate of the flies challenged with *Diaporthe* FY. Dechlorinated eggs were used to generate a specific interaction between hosts and specific microbes as previously described [23]. The results presented in Fig. 2a show that the hatched larvae rapidly succumbed to *Diaporthe* FY infection in vials containing more than 4 × 10^6^ CFU spores. The eclosion duration of the flies infected with *Diaporthe* FY was extended compared to conventionally reared (CR) flies (Fig. 2a). This finding implies that *Diaporthe* FY impeded the normal development of *Drosophila*. Consistent with a previous study [24], *Drosophila* was susceptible to *Aspergillus flavus* (Additional file 1), suggesting that the pathogenic fungus-induced morbidity of *Drosophila* could be generated in our laboratory. Intriguingly, the developmental time of adults challenged with less than 4 × 10^6^ CFU spores was shorter than that of germ free (GF) flies (Fig. 2a). This can be partially explained by the fact that the fungi could produce very low concentrations of toxic secondary metabolites during the exponential phase of nutritional growth; thus, its growth-promoting effects could override the inhibition of host development. Additionally, the survival of CR adults fed the *Diaporthe* FY molds was significantly lower than that of the mock-infected flies (Fig. 2b). Due to the causal relation between GF hosts and microbes, the survival of GF adults subjected to *Diaporthe* FY was also lower than that of their counterparts (Fig. 2b). These results suggest that *Diaporthe* FY diminished the relative survival of the flies. Moreover, innate immunity-associated genes were significantly triggered in the *Diaporthe* FY-infected flies compared to their counterparts (Fig. 2c), indicating that the flies developed a robust immune response to this invader. In agreement with a previous study [25], immune deficient *PGRP-LC* mutant flies were much more susceptible to *Diaporthe* FY than the wild-type flies (Fig. 2b), indicating that *Diaporthe* FY was a potentially virulent pathogen towards *D. melanogaster*. Hence, we subsequently investigated the survival to septic injury by injecting *Diaporthe* FY spores into the body cavity of flies. Concomitantly, the flies challenged with septically infection were more likely to die compared to their corresponding control flies (Fig. 2d), indicating that *Drosophila* was susceptible to *Diaporthe* FY. Collectively, these results suggest that *Diaporthe* FY functions as a *Drosophila*-associated pathogen.

### *L. plantarum* undermines the susceptibility of *Drosophila* to *Diaporthe FY* infection

Given that pathogenic fungal infections impose morbidity and mortality upon animals in the wild, it was proposed that the natural host microbiota could promote the survival of flies challenged with *Diaporthe* FY [26]. Previous studies have shown that *L. plantarum*, due to its vast metabolic repertoire, fostered host development by accelerating their growth rate [17]. We then examined the antifungal response of *L. plantarum* against *Diaporthe* FY by simultaneously inoculating them into sterilized *Drosophila* GF eggs. Indeed, supplementation with *L. plantarum* efficiently rescued the lethality of the *Diaporthe* FY-infected flies, as well as ameliorated the delay of pupa formation and adult eclosion (Fig. 2e and f). This result suggests that *L. plantarum* mitigated *Drosophila* susceptibility to *Diaporthe* FY.

### *L. plantarum* suppresses the growth of *Diaporthe* FY

**Fig. 3:**
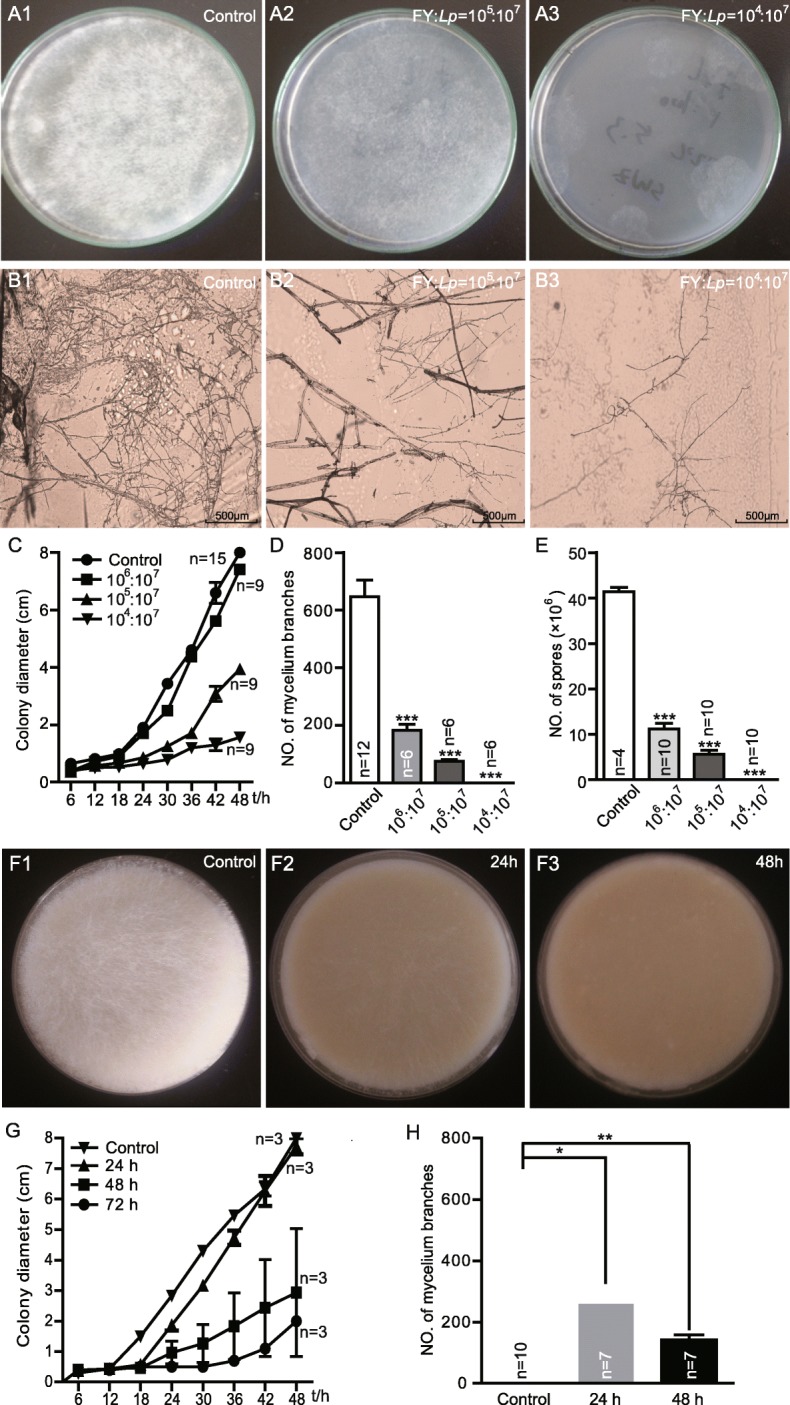
*L. plantarum* hinders the growth of *Diaporthe* FY in vitro*.* **a** *L. plantarum* inhibited the growth of *Diaporthe* FY in a dose-dependent manner. Both *Diaporthe FY* and *L. plantarum* (at different ratios: A1, *Diaporthe* FY; A2, 1:100; A2, 1:1000) were simultaneously inoculated into nutrient rich medium and incubated at 25 °C for 24 h. The growth of *Diaporthe* FY is shown. **b** The growth of mycelia was inhibited in the case of *L. plantarum*. **c-e** The quantification of the colony growth rate, the number of mycelia, and spores of *Diaporthe* FY. **f** The growth of *Diaporthe* FY in fly food pre-incubated with *L. plantarum*. White mycelia were observed on the surface of the food (F1), whereas fewer mycelia were observed in *L. plantarum*-treated food (F2 and 3). **g** The colony growth and number of mycelium branches of *Diaporthe* FY in medium pre-incubated with *L. plantarum*. The one-sample *t*-test; * *P* < 0.05; ** *P* < 0.01

To confirm that *L. plantarum* competes with *Diaporthe* FY, we tested the inhibition of *L. plantarum* on the growth of *Diaporthe* FY in vitro. Our data showed that *L. plantarum* outcompeted *Diaporthe* FY in a dose-dependent manner (Fig. 3a). We quantified the suppressive effects by colony growing, mycelia branching, and spore forming assays. First, the colony growth of *Diaporthe* FY was decreased by *L. plantarum* compared to the control (Fig. 3b and c). Secondly, there were fewer mycelia in the presence of *L. plantarum* (Fig. 3d). In addition, the number of spores was dramatically decreased following *L. plantarum* inoculation (Fig. 3e). Taken together, these results suggest that *L. plantarum* potently reduced the survivability of *Diaporthe* FY.

We further speculated that after dominating the niche, *L. plantarum* could thwart *Diaporthe* FY colonization. To this end, we pre-incubated the food with *L. plantarum* for different lengths of time (24 h, or 48 h) and added *Diaporthe* FY to the “modified” diet. In agreement with the simultaneous competition, the growth of *Diaporthe* FY was also hindered when it was pre-inoculated into the food with *L. plantarum.* Of note, the longest incubation period completely inhibited the growth of *Diaporthe* FY (Fig. 3f). This inhibitory effect was further fortified by the decreased number of mycelia and spores (Fig. 3g and h). Taken together, these findings support the *L. plantarum-*mediated inhibition of *Diaporthe* FY growth and dispersal.

### Lactic acid inhibits the growth of *Diaporthe* FY

**Fig. 4:**
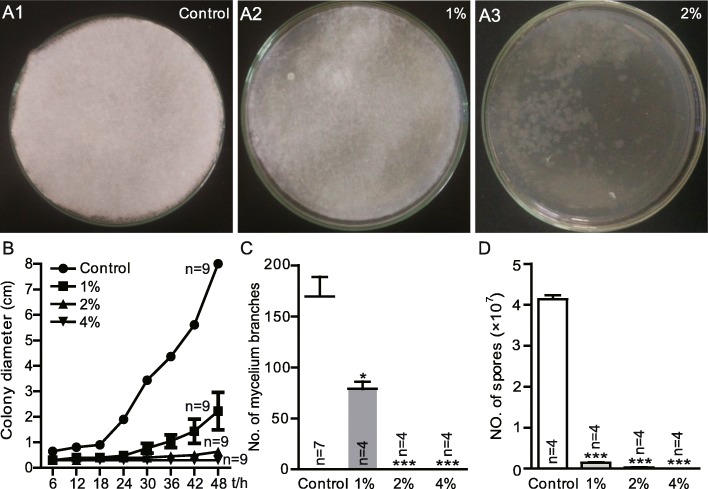
Lactic acid exerts anti-fungal properties. **a** The growth of *Diaporthe* FY at different concentrations of lactic acid. *Diaporthe* FY was inoculated into nutrient-rich medium containing different concentrations of L-lactate, and incubated at 25 °C for 48 h. **b** The colony growth rate of *Diaporthe* FY treated with lactic acid. **c** and **d** The number of mycelia and spores in the case of lactic acid. The one-sample *t*-test; NS *P* > 0.05; * *P* < 0.05; ** *P* < 0.01; *** *P* < 0.001

To further characterize the mechanism involved in *L. plantarum-*mediated inhibition of *Diaporthe* FY, we next sought to identify candidate inhibitory factors derived from *L. plantarum* metabolites. Lactic acid is generated by many lactic acid bacteria and exerts its antimicrobial effects by disrupting the cytoplasmic membrane or reducing the intracellular pH [27]. Since the strain of *L. plantarum* used in this study typically produced more than 75 mM (approximately 0.7% w/v) L-lactate at the end of fermentation (Additional file 2), we therefore focused on the role of L-lactate on inhibiting the growth of *Diaporthe* FY. To determine whether lactic acid could inhibit the growth of *Diaporthe* FY, we scored the fungal growth on a medium supplemented with different concentrations of L-lactate. The results showed that the growth of *Diaporthe* FY was inhibited in a L-lactate dose-dependent manner (Fig. 4a). *Diaporthe* FY was modestly inhibited by 0.5% lactic acid and robustly inhibited by 1% or higher doses of lactic acid. It was unlikely that this antifungal property was derived from the lower pH value, since the comparable pH values adjusted with HCl were unable to inhibit the growth of *Diaporthe* FY (Additional file 3). These data indicate that the inhibition of *Diaporthe* FY was partly attributed to the properties of lactic acid. The data further showed that the colony growth of lactic acid-treated *Diaporthe* FY was prominently decreased compared to the mock-infected flies (Fig. 4b). Likewise, the number of mycelia and hyphae were considerably lowered in the presence of lactic acid (Fig. 4c and d). Overall, our data suggest that lactate was an important factor that could inhibit the growth of *Diaporthe* FY.

### The synergism between *Drosophila* and *L. plantarum* to combat *Diaporthe* FY infection

**Fig. 5:**
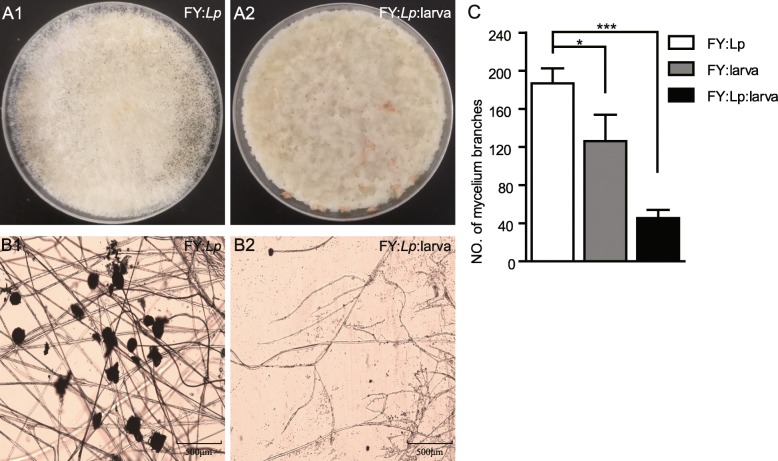
*Drosophila* and *L. plantarum* synergize to defend against *Diaporthe* FY. **a** The colony growth of *Diaporthe* FY with *L. plantarum* in the presence or absence of larvae. A total of 25 third instar larva were transferred to each plate with a fly diet inoculated with *Diaporthe* FY, *L. plantarum,* or both. The plates were incubated at 25 °C and colony growth was assessed at 72 h. **b** Mycelia of *Diaporthe* FY with *L. plantarum* in the absence or presence of larvae. **c** The number of branching mycelia in the absence or presence of larvae. The one-sample *t*-test; *** *P* < 0.001

Upon pathogenic infection, *Drosophila* initiates an innate immune response through the production of reactive oxygen species and antimicrobial peptides. It was assumed that collaboration between the host and its commensals could more efficiently resist pathogenic fungi than either alone. To the end, the early third-instar larvae were seeded into the fly diet with *Diaporthe* FY and *L. plantarum*. Our data revealed that the colony growth of *Diaporthe* FY was significantly obstructed in the presence of larvae compared to that in the absence of larvae (Fig. 5a and c). This result indicated that *Drosophila* and commensals collaborated to antagonize pathogens. Similarly, the number of branching mycelia was reduced in the presence of larvae compared to jn the absence of larvae (Fig. 5b and c). Intriguingly, *Diaporthe* FY did not form any spores in the case of larvae, partly due to the disrupted configuration of the hypha. These results demonstrate that *Drosophila* synergized with *L. plantarum* to suppress the growth of *Diaporthe* FY, which was critical for host survival against infection.

### *L. plantarum* reverses ovipositional avoidance to *Diaporthe* FY

**Fig. 6:**
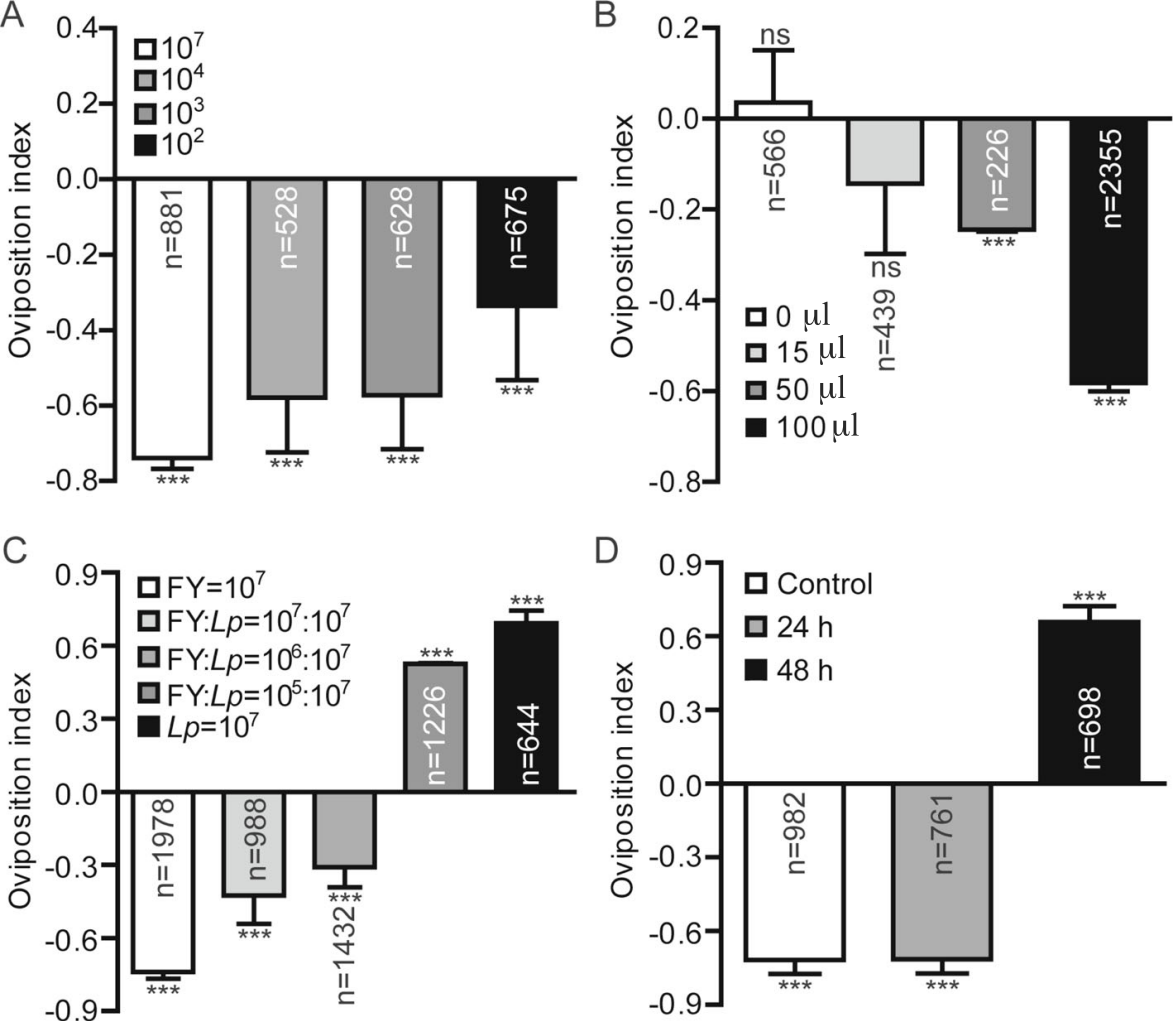
*L. plantarum* prevented oviposition avoidance towards *Diaporthe* FY. **a** Quantification of egg-laying avoidance to diet fermented with *Diaporthe* FY. Egg-laying preference was assayed using a two-choice chamber. Mated females were transferred to the chamber and allowed to lay eggs for 8 h. The number of eggs was counted on each half, and the oviposition preference was calculated. **b** Ovipositional avoidance towards metabolites of *Diaporthe* FY. The supernatant of liquid fly food was evenly distributed on the surface of halves in a dose-dependent manner. **c** *L. plantarum* reduced oviposition avoidance towards *Diaporthe* FY. **d** Oviposition preference for *Diaporthe* FY-treated diet that was previously inoculated with *L. plantarum*. The one-sample *t*-test was used to assess the mean deviance of each column from 0, *n* = 6–14; NS *P* > 0.05; * *P* < 0.05; ** *P* < 0.01; *** *P* < 0.001

Using various sensory modalities, animals are able to swiftly respond to certain stimuli in their surrounding environment. To enhance the survival and fitness of their offspring, *Drosophila* females select favorable sites to deposit their eggs [28, 29]. Our previous work showed that commensals (e.g., *L. plantarum*), elicited an oviposition preference of *Drosophila* using the two-choice assay [23]. Since *Diaporthe* FY imposed morbidity on both larval and adult *Drosophila* (Fig. 2), it would be reasonable to hypothesize that *Drosophila* could sense the presence of *Diaporthe* FY in potential egg-laying sites. As expected, female adults overwhelmingly avoided egg-laying on the food treated with *Diaporthe* FY (Fig. 6a). Many molds produce an extraordinary range of secondary metabolites that repel insects [30, 31]. Indeed, the flies were robustly repulsed to laying their eggs on the surface of the halves containing metabolites of *Diaporthe* FY (Fig. 6b), which indicated that secondary metabolites of *Diaporthe* FY alerted the flies to the presence of toxic molds. We next wondered whether *L. plantarum* could alter the ovipositional repulsion of females to *Diaporthe* FY. As expected, the addition of *L. plantarum* dose-dependently increased the oviposition index of the females, and even switched to laying eggs in fermented food with a predominance of *L. plantarum* (Fig. 6c), indicating that *L. plantarum* attenuated ovipositional avoidance to *Diaporthe* FY. We further asked whether *L. plantarum* could abolish the ovipositional aversion to *Diaporthe* FY when it had dominated the community. The diet was pre-incubated with *L. plantarum* for different lengths of time and then exposed to *Diaporthe* FY. We found that although the flies were aversive to ovipositing in fermented food pre-incubated with *L. plantarum* for 24 h, this response was over-ridden in fermented food pre-incubated with *L. plantarum* for 48 h (Fig. 6d). Hence, our results demonstrated that commensals, if dominating the niche, significantly reversed the oviposition avoidance to pathogenic fungi.

## Discussion

Animals are colonized by abundant and diverse microbiota, which affect many aspects of host physiology and pathology [32, 33]. Despite advances in sequence-depended microbial profiling, little is known about the role of mutualistic microbes in antagonizing fungal pathogens in their host animals. This study has shown that *Drosophila*-associated commensal bacteria exhibited inhibitory capabilities against fungal infections. Moreover, commensal *L. plantarum* suppressed the growth of *Diaporthe* FY in vitro, and mitigated the fungal toxicity to *Drosophila* in vivo. In particular, *L. plantarum* predominantly overrode the egg-laying avoidance of *Drosophila* to *Diaporthe* FY. This integrative and synthetic community of *Drosophila,* bacterium, and fungus provides insight into the fundamental concepts and precise mechanisms involved in animal-commensal-pathogen interactions. The infection-associated *Drosophila* model used in this study was previously established by injecting a lethal dose of the pathogens into the body cavity of adult flies [34]. However, little attention has been devoted to complex bacterial context. In fact, a bacterial consortium approach that views the microbiome as a set of functional traits is likely to offer a more comprehensive means of protecting hosts from threatening pathogens. Consistently, the observed phenomenon supports our hypothesis that the protective traits conferred by the host’s natural microbiota were naturally selected to enhance host survival in the context of a challenging environment rife with pathogens. Thus, our approach should facilitate the development of animal models that can better recapitulate complex natural pathological phenomena.

Entomopathogenic fungi play a pivotal role in regulating insect populations in nature [12]. These fungi have evolved highly diversified lifestyles, and are in competition with insects for natural resources. Metagenetic analysis has unraveled dozens of secondary metabolic gene clusters that encode an astonishing variety of secondary metabolites [10, 30, 31]. Although pathogenic fungi seriously threaten the survival of flies in nature, animals have developed fundamental antifungal strategies to thrive in the world. *Drosophila* possess an innate immune system that induces the production of antimicrobial peptides and reactive oxygen species to prevent fungal infection [35]. Alternatively, the mitigation of fungal toxicity can also be attributed to complex interactions between hosts and microbes. Commensals and/or probiotics outcompete pathogens through chemical inhibition, physical and nutritional competitive exclusion, and a variety of other adaptive mechanisms [36]. Lactic acid bacteria are widely considered to be natural antifungal microbes that can be found in fermented substrates [37, 38]. Lactic acid bacteria hamper the growth of many pathogenic fungi by inhibiting their adherence, establishment, replication, various pathogenic actions, and can also decompose mycotoxin to a certain extent [39]. In turn, larvae-derived maintenance factors enhance the propagation of this bacterial population, and override this cost of feeding and gut transit, forming an inextricable holobiont [26]. Notably, *L. plantarum* mon-associated and GF flies usually more resist against pathogens than CR ones. This is in part explained by the finding that CR flies are prone to dysbiosis with an increase in intestinal bacterium loading, and dramatic changes in the components and functions of the microbiota, which could aggravate the pathogenesis. More importantly, adult *Drosophila* function as a vector, and promote the ongoing dispersal of bacteria in the environment [40]. Therefore, the synergistic interaction between *Drosophila* and the microbiota exerts antifungal activity against a broad spectrum of molds in the wild.

While hosts exhibit behavioral-immune responses against pathogens, this important function remains underappreciated [41]. Female *Drosophila* possess the innate behavior of selecting favorable oviposition sites to increase the survivability of their offspring. Since larvae are vulnerable to predators due to their restricted mobility, selecting a favorite site to lay their eggs is an innate behavior of female. The hypothesis of ‘mother-knows-best’ ensures that female egg-laying decisions have evolved to identify locations that will promote the greatest survival of offspring [42]. This is achieved by evaluating the nutritional and microbial content of potential oviposition sites. Previous studies, including our work, have shown a general theme that *Drosophila* are robustly allured to lay eggs in fermented food by commensal *Lactococcus*, *Lactobacillus*, *Weissella,* and *Saccharomyces* [23, 43]. However, they were vigorously repelled by the presence of harmful molds (e.g., *Penicillium expansum*) [44]. Thus, the females avoid laying eggs on the sites with fungal toxicants, which efficiently protects the hatched larvae from infection. Survival and reproduction strategies should be employed in the context of systemic ecology, in which flies balance the benefits and threats of commensals and pathogens, respectively. Therefore, *Drosophila* distinguishes commensals from pathogens and selects favorable sites for egg-laying. It is conceivable that females still switch to laying-eggs on fermented food when the nutrition of commensals overrides threats from pathogens. This is consistent with the observation that fermented food with *L. plantarum* and *Diaporthe* FY still attract females to lay eggs, partially because *L. plantarum* has dominated the niche and outcompeted *Diaporthe* FY.

Utilizing the *Drosophila* model system, we have revealed an ecological phenomenon whereby indigenous microbiota are required to defend *Drosophila* against pathogenic fungal infection. This model provides a reductionist approach for disentangling the inherent complexity of host-microbe interactions from the organismic to the molecular level. A more complete understanding of the underlying mechanism of the host and bacterial response to pathogens will facilitate the discovery of innovative probiotic interventions to foster the fitness of the microbe-host holobiont.

## Conclusion

In this study, we identified and characterized *Diaporthe* FY as a potential *Drosophila* pathogen*.* Commensal *L. plantarum* is required and sufficient to ameliorate the susceptibility of *Drosophila* to *Diaporthe* FY by generating lactic acid. *L. plantarum* counteracted the avoidance of oviposition *on Diaporthe FY*-associated substrates, which enhances the fitness of *Drosophila* offspring. In summary, our findings provide a first insight into the natural interplay between *Drosophila*, commensals and pathogens.

## Methods

### *Drosophila* and microbe husbandry

The Oregon R strain of *D. melanogaster* was used as wild-type flies. PGRP-LC mutant flies were kindly gifted by Dr. Zhai (Hunan Normal University, China). All flies were reared at 25 °C, 60% humidity with a 12 h/12 h light/dark cycle on standard cornmeal-yeast-sucrose food unless otherwise stated [23]. *Drosophila* was cultured with standard cornmeal-sugar-agar medium (1 L) (1350 mL ddH_2_O, 13 g agar, 0.83 g CaCl_2_, 31.6 g sucrose, 63.2 g glucose, 77.7 g cornmeal, and 24 g yeast power) [28]. Fungi were cultured using Potato Dextrose Agar (PDA) medium at 25 °C. *L. plantarum* were cultured in selective medium De Man Rogosa Sharpe (MRS) at 35 °C. The mixture of *Diaporthe* FY and *L. plantarum* was cultured in Mueller-Hinton Agar (MHA) medium at 30 °C. *L. plantarum* was isolated from the gut of *Drosophila* with the Genbank accession number, KY038178. *Aspergillus flavus* (3.3950) was obtained from the China General Microbiological Culture Collection Center.

### Fungi isolation and identification

Fungal strains were isolated from fly food with molds using PDA medium. Mycelium was briefly picked up, transferred to PDA medium, and incubated for 48 h at 25 °C. This procedure was repeated five times for purification. The fungus was grown on liquid PDA medium for 2 d at 25 °C. The mycelium was collected, and the genomic DNA was extracted and purified using a DNA isolation kit (Tiangen, Beijing, China). For identification, the internal transcribed spacer regions (ITS1 and ITS2) were amplified using PCR (Thermocycler, Germany) with the universal ITS primers, ITS1 (5′ -TCC GTA GGT GAA CCT GCGG-3′) and ITS4 (5′-TCC TCC GCT TAT TGA TAT GC-3′). The PCR products were sequenced by a commercial company (Shenggong, Shanghai). The ITS DNA sequences were aligned against the nucleotide–nucleotide database (BLASTn) of the National Center for Biotechnology Information (NCBI) for the final identification of the isolates.

### Colony growth, branching mycelium, and sporulation assay

A 1 μL extract with 10^6^ spores was inoculated into MHA medium. To assess the effect of acid on fungi, L-lactate or chloric acid was directly added to the medium in a dose-dependent manner. The diameter of the fungal colony was measured at the 6 h interval. Sterile coverslips were inserted into the agar medium at an angle of 45°, and the number of branching mycelia on the coverslip was scored using a microscope. To examine the number of spores on the medium, all mycelia growing on the surface of the medium were transferred to 10 mL PBS. The medium was violently shaken, and 100 μL of the medium was transferred to the hemocytometer to count the spores under a microscope.

### Germ-free and gnotobiotic flies

The process of generating germ free (GF) embryos was performed similar to that previously described [15, 23]. Eggs laid on grape juice agar media were collected within 8 h and rinsed with ddH2O to remove the yeast paste on the surface. Next, the eggs were sequentially subjected to 1:30 diluted Walch sanitizer (Procter & Gamble Co., Cincinnati, OH, USA), 2.5% hypochlorite sodium for 1 min (Sigma Aldrich, St. Louis, MO, USA), 70% ETOH for 1 min, and finally PBS containing 0.01% TritonX-100. The absence of bacteria was routinely confirmed by PCR analysis of 16S rRNA primers (8FE and 1492R) on fly homogenates and culturing the homogenates in Lysogeny Broth (LB)-agar plates. Sterilized GF eggs were transferred to vials with autoclaved media within a biosafety cabinet. GF flies were supplemented with either unknown or known bacteria to generate conventionally reared (CR) or gnotobiotic flies.

### Survival rate and developmental timing of flies

For the survival test, 30 eggs were transferred to vials with casein-cornmeal-agar medium within 10 h after egg-laying. Eggs were exposed to fungi or bacteria, and the survival ratio was calculated. The number of pupae and adult formation was recorded, and the formula used to calculate developmental timing was expressed as:

T = (T1 × N1 + T2 × N2 + … + Tm × Nm)/ (N1 + N2 + … + Nm).

where T represents the developmental timing, T_m_ is the days after egg laying, and N_m_ is the number of pupae and adults on the T_m_ day [15, 23].

### Oral and injury infection

For survival assay, 20 male and female flies following 5 d after eclosion were collected into each vial. For oral infection, flies were transferred to vials that were pre-inoculated with 10^8^ spores and incubated at 25 °C for 48 h. Systemic infections (septic injury) were performed by pricking the thorax of adult females with a thin needle previously dipped into a concentrated pellet of bacterial culture or into a suspension of *Diaporthe* FY spores [45]. All of the infected flies were incubated at 25 °C. At least three tubes containing 20 flies were used for survival experiments and the survival count was scored daily.

### Real-time quantitative PCR analysis

Male adult flies were fed *Diaporthe* FY for 24 h. Fly guts were dissected in cold PBS buffer, and the total RNA was extracted with TRIzol reagent (Invitrogen, USA). Up to 2 mg of total RNA was used as a template for reverse transcription with the oligo-dT primer for real-time quantitative PCR (BioRad). The primer sets for ATT, Dpt, and Duox were as previously described [35]. The ΔCt method was employed to analyze the data using *rp*49 as the reference gene. The relative expression value was calculated with formula: △Ct = Ct (target gene) - Ct (reference gene), the relative = 2^-△△Ct^.

### Oviposition preference assay

Two-choice oviposition chambers were constructed in a similar manner as described in previous studies [23, 28]. In each chamber, the flies were able to choose their oviposition sites between two types of fermented substrates. To create the fermented substrates, food agar was sterilized by autoclaving at 121 °C, and plated with either 100 μL of *Diaporthe* FY, *L. plantarum,* or ddH_2_O for the controls. Flies were then incubated at 25 °C for 48 h. To assemble the oviposition chamber, a razor blade was used to divide the agar into halves, and two different oviposition substrates were hand-puzzled into a Petri dish. To assess the fungal metabolites, liquid fly food (without agar) was inoculated with *Diaporthe* FY and incubated at 25 °C for 48 h. Fermented fly food was centrifuged at 12,000 rpm for 15 min, and the supernatant was distributed onto the surface of fly food in each of the two-choice oviposition chambers. A total of 50 female flies were collected and mated for 6 h after transfer to the device. Finally, the flies were removed and the number of eggs on each half of the two-choice chamber was counted, and the oviposition index (OI) was calculated using the following equation: OI = (no. of eggs laid on experimental food - no. of eggs laid on control food)/total no. of eggs laid.

## Supplementary information

**Additional file 1.** Pathogenic fungi undermine the fitness of D*rosophila*.

**Additional file 2.*** L. lactobacter* generates lactic acid overtime.

**Additional file 3.** Modest pH decrease exhibits a trivial role in inhibiting the growth of *Diaporthe* FY.

## Data Availability

The dataset for the current study is available from the corresponding author upon reasonable request. *L. plantarum* was isolated from the gut of *Drosophila* with the Genbank accession number: KY038178.

## Abbreviations

CFU: Colony formation unit
CR: Conventionally reared
GF: Germ free
OI: Oviposition index
