# Supplementary figures and images for: Pathogenic fungi-induced susceptibility is mitigated by mutual Lactobacillus plantarum in the Drosophila melanogaster model
Source: BMC Microbiol. 2019 Dec 21;19:302. doi: 10.1186/s12866-019-1686-1 (PMC6925846; doi:10.1186/s12866-019-1686-1)

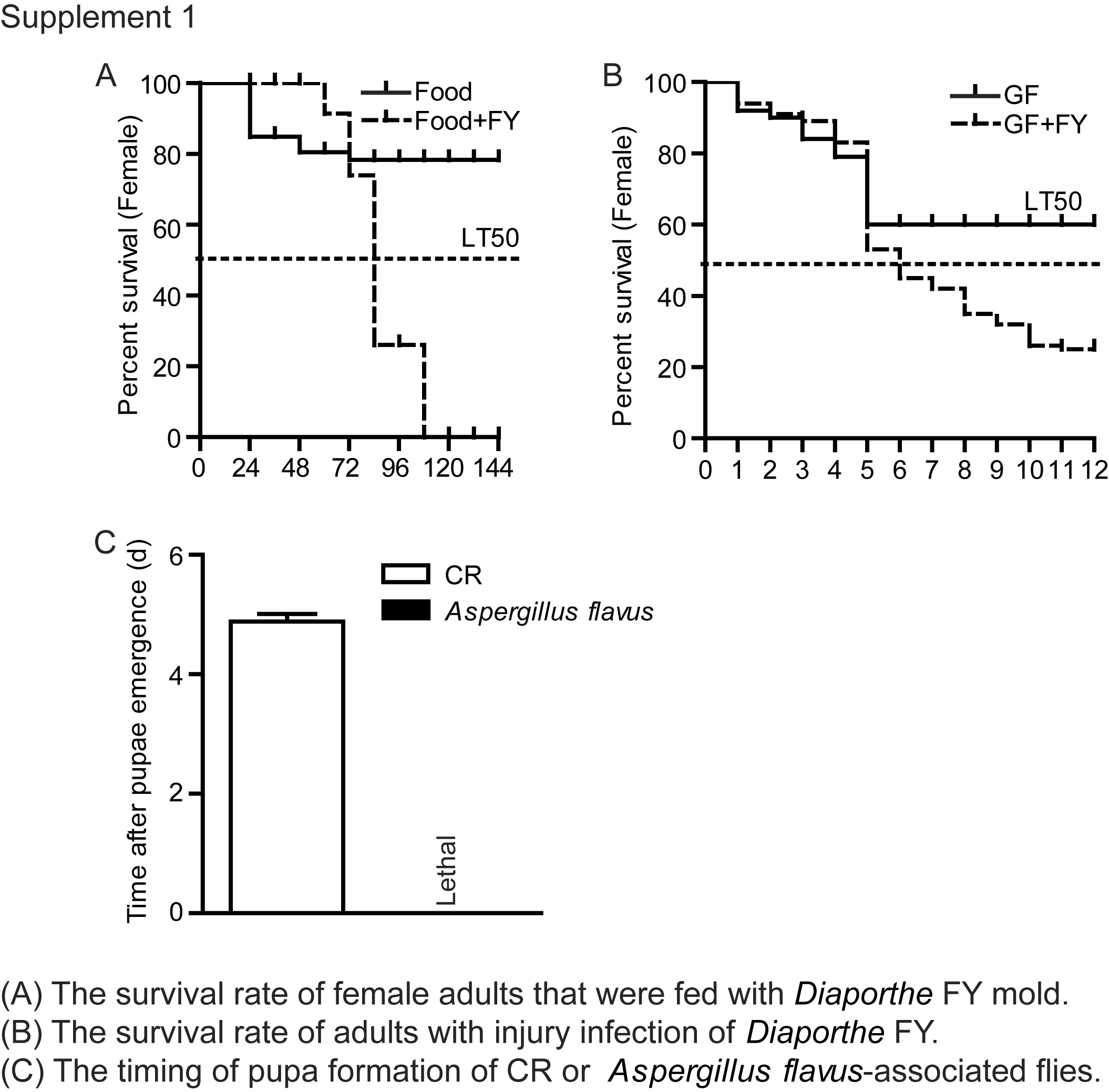

Supplement: Supplementary file 1 — Additional file 1. Pathogenic fungi undermine the fitness of Drosophila. [file 12866_2019_1686_MOESM1_ESM.tif]

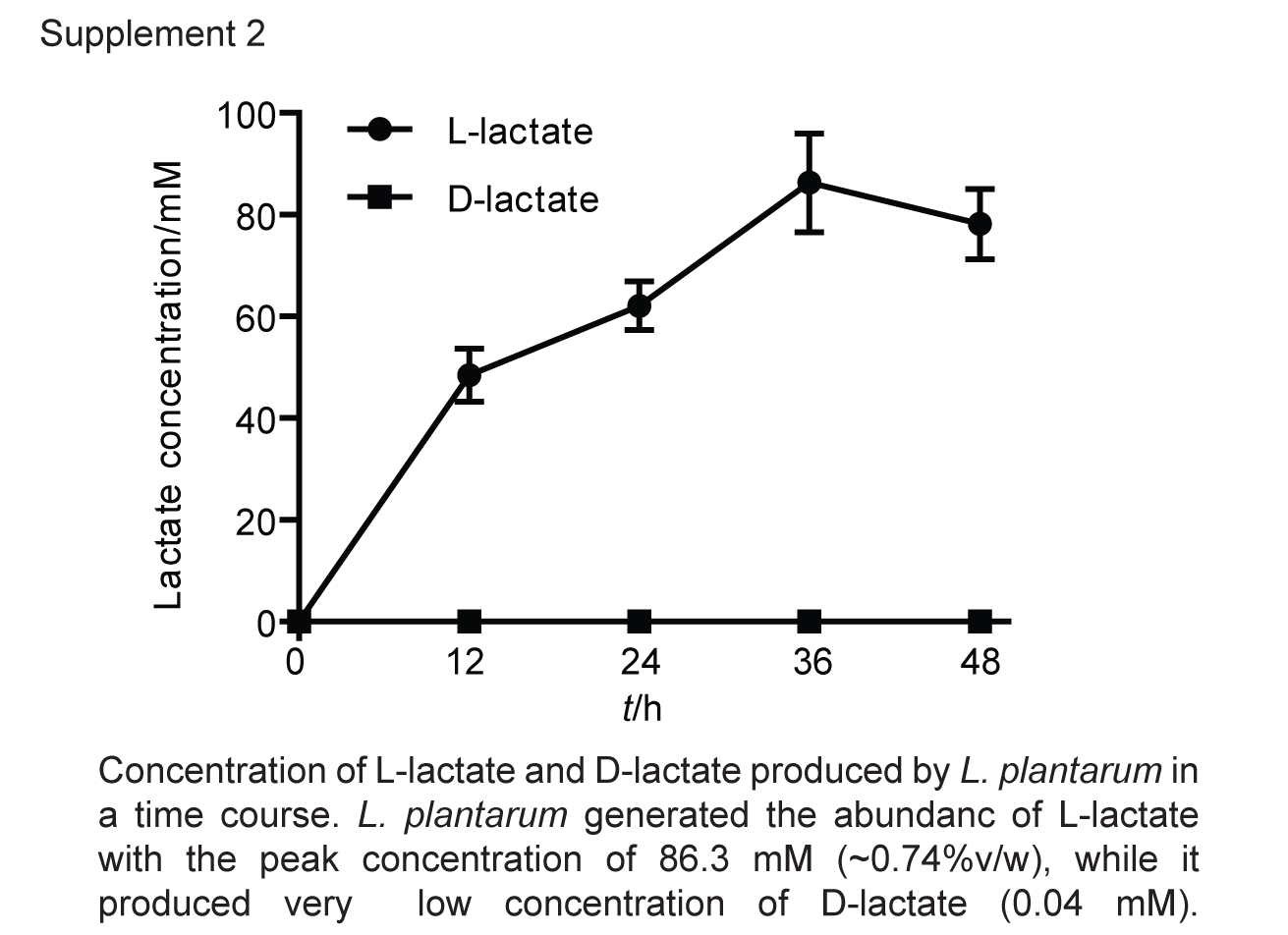

Supplement: Supplementary file 2 — Additional file 2. L. lactobacter generates lactic acid overtime. [file 12866_2019_1686_MOESM2_ESM.tif]

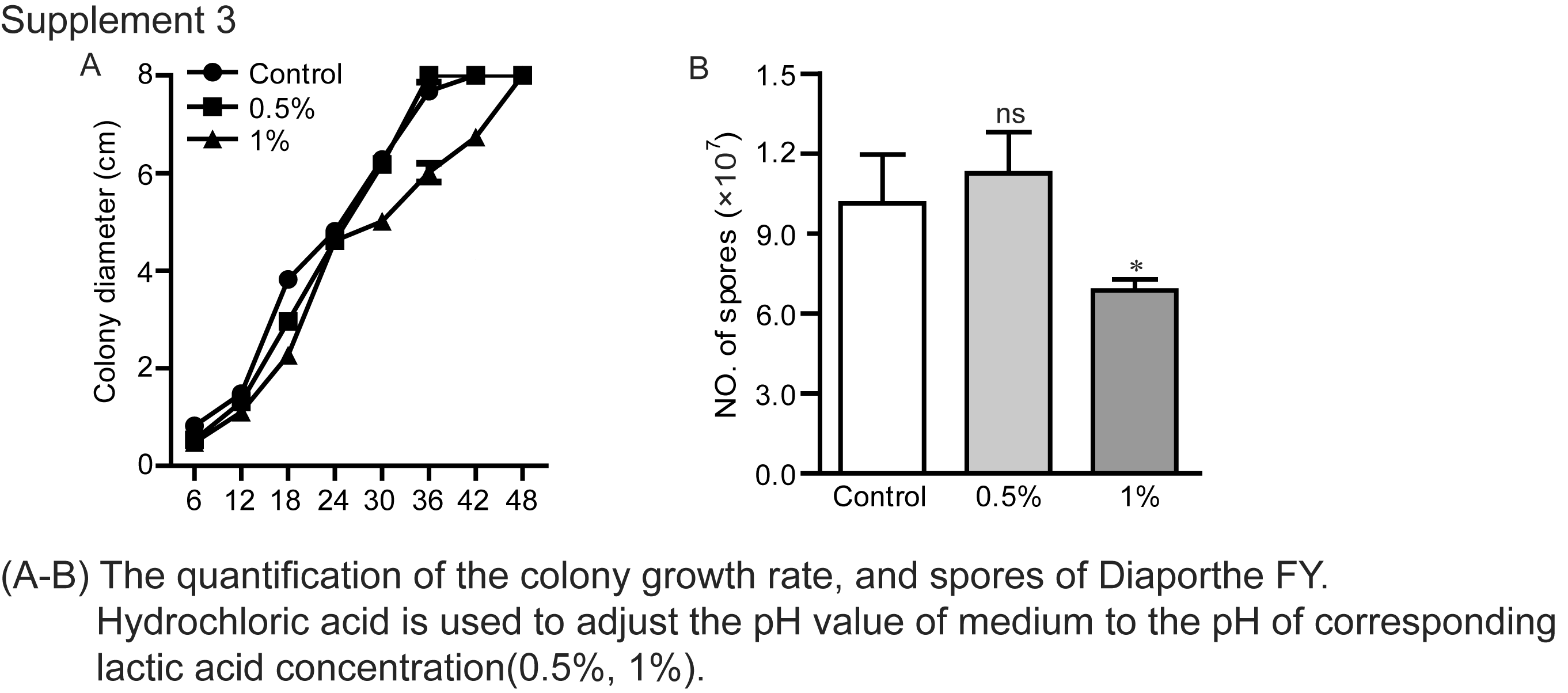

Supplement: Supplementary file 3 — Additional file 3. Modest pH decrease exhibits a trivial role in inhibiting the growth of Diaporthe FY. [file 12866_2019_1686_MOESM3_ESM.tif]
